# Platinum-Functionalized Graphene Oxide: One-Pot Synthesis and Application as an Electrocatalyst

**DOI:** 10.3390/ma16051897

**Published:** 2023-02-24

**Authors:** Anisoara Oubraham, Daniela Ion-Ebrasu, Felicia Vasut, Amalia Soare, Ioan-Sorin Sorlei, Adriana Marinoiu

**Affiliations:** National Institute for Cryogenics and Isotopic Technologies ICSI-Rm. Valcea, ICSI Energy, Uzinei Str. no. 4, 240050 Ramnicu Valcea, Romania

**Keywords:** pH changing, microwave reduction, catalyst, platinum, graphene oxide

## Abstract

This paper presents the preparation of platinum on a reduced graphene oxide matrix (PtrGO) using the microwave-assisted method with three different pH solutions. The platinum concentration determined by energy-dispersive X-ray analysis (EDX) was 4.32 (weight%), 2.16 (weight %) and 5.70 (weight%), corresponding to pH 3.3, 11.7 and 7.2, respectively. Pt functionalization of reduced graphene oxide (rGO) decreased the rGO specific surface, as shown by Brunauer, Emmett and Teller (BET) analysis. An XRD spectrum of platinum-decorated reduced graphene oxide (rGO) showed the presence of the associated phases of rGO and centered cubic platinum peaks. An oxygen reduction reaction (ORR) electrochemical characterization performed using the rotating disk electrode (RDE) method showed that in PtGO1 synthetized in an acidic environment, with 4.32 Pt (weight%) determined by EDX, platinum is much more dispersed, which explains its better electrochemical oxygen reduction reaction performance. Koutecky–Levich (K-L) plots calculated at different potentials prove a good linear relationship. Electron transfer numbers (*n*) determined from the K-L plots are between 3.1 and 3.8, which confirms that the ORR for all the samples can be regarded as first-order reaction kinetics of O_2_ concentration formed on the Pt surface during ORR.

## 1. Introduction

Energy sustainability and conversion are serious issues in the modern world. The state-of-the-art fuel cell will play an important role in reducing coal energy dependency while reducing pollution [1]. Fuel cells powered by hydrogen are considered the ideal solution for non-polluting portable (cell phones, laptops and smart gadgets), mobile and stationary applications. The proton exchange membrane fuel cells (PEMFCs) generate electrical current by electrochemical hydrogen oxidation at the anode and oxygen reduction at the cathode. The protons released during the oxidation of hydrogen are conducted through the proton exchange membrane to the cathode. As the membrane is not electrically conductive, the electrons released from the hydrogen travel along the electrical detour provided, and an electrical current is produced [2]. The oxygen reduction reaction (ORR) is a key elemental reaction that occurs in fuel cells, where oxygen is reduced to water at the cathode. The ORR is considered to be six or more orders slower [3,4] than the hydrogen evolution reaction (HER) at the anode. Therefore, ORR is the limiting step in the overall performance of fuel cells and has a crucial role in determining the efficiency and power of the cell. The ORR is complex and involves multiple intermediate species and reaction pathways. However, the ORR mechanism can generally be summarized as a four-electron transfer process, as follows: (i) electron transfer to O_2_ to form superoxide ion (O_2_)^−^; (ii) proton (H^+^) transfer to O^2−^ to form hydrogen peroxide (H_2_O_2_); (iii) electron transfer to H_2_O_2_ to form water (H_2_O) and hydroxyl radicals (OH^−^); and (iv) H^+^ transfer to OH^−^ to form H_2_O. The exact mechanism and pathways of the ORR can vary depending on the conditions and the type of catalyst used, but the overall goal is to transfer four electrons from the electrode to oxygen molecules to produce water. Today, commercial fuel cells use noble metals (e.g., platinum, ruthenium and iridium) supported on carbon black-type conductive materials (e.g., Vulcan XC-72, Ketjen black EC 300J and Ketjen black EC 600JD). However, during the oxygen reduction reaction (ORR) that takes place at the cathode, carbon black is electrochemically corroded, which encourages the scientific community to find innovative materials that can offer better solutions, one of these being graphene-based materials [5].

Graphene, together with graphite, diamond and fullerenes, is an allotrope form of carbon formed by covalently bonded carbon atoms grouped in a planar hexagonal lattice. Graphene has a bidimensional (2D) structure, with sp^2^ hybridization and a one-atomic-layer thickness [6,7,8]. A monolayer of graphene has high mechanical strength (about 130 GPa) [4], is very light (0.77 mg/m^2^) and is elastic and more flexible than carbon fiber [9,10]. Graphene is a semimetal or zero-gap semiconductor with an electron hole symmetric distribution in two circular cones linked at one point (Dirac cones) [11]. This unique electronic structure confers an intrinsic electronic mobility > 10000 cm^2^·V^−1^·s^−1^ upon graphene [12,13]. Based on these properties, graphene can be considered an ideal base for catalysts, electronics, energy storage devices, sensors, transparent electrodes, super capacitors and fuel cells [14,15,16,17,18]. Graphene is regarded as an alternative to the commercial carbon black catalyst supports due to its outstanding electrical conductivity, chemical stability, electrochemical durability and remarkably high specific surface area (2600 m^2^/g) [19].

Graphene oxide (GO) is a 2D multilayer graphene-based material with oxygen-functionalized side groups, e.g., epoxide and hydroxyl on the basal plane and carbonyl and carboxy at the edges, with the latter being considered the most reactive for GO functionalization [20]. The presence of the polar oxygen-based functional groups increases the hydrophilic properties of the graphene oxide and makes it more dispersible in polar organic solvents and water. Therefore, GO can form well-dispersed inks and solutions, for which reason it is recommended for utilization as a catalytic support for fuel cells, electrolysis, batteries, sensors and other applications.

Moreover, GO functionalization can be carried out through different methods, such as covalent surface modification [21], noncovalent functionalization [22] and doping [23]. Typically, graphene oxide functionalization with platinum-based nanoparticles is carried out using hydrothermal techniques [24,25], ethylene glycol (EG) reduction [26] and solvothermal methods [27]. The microwave-assisted method is one of the recent techniques for nanostructured catalyst synthesis formed by graphene oxide substrate functionalized with precious metals (e.g., Pt, Au, Ru or Ir) [28,29], transitional metals (e.g., Co, Cr, Ni, Fe, etc.) [30,31] or metal-free platinum nanostructured catalysts for fuel cell applications [32]. The advantage of preparing nanostructured catalysts using the microwave method consists in fast, homogeneous heating resulting from the direct interaction of the microwave radiation with the solvent molecules and the reducing agent. The microwave method can achieve a short nucleation and crystallization time, resulting in small-sized particles and high dispersion [28]. The advantages of using microwaves, as compared to traditional heating methods, are uniform heating, high speed and energy efficiency. Moreover, the simplicity of the equipment and the short reaction time render this technique energy-saving and allow it to meet economic considerations [4,32]. During the microwave process, platinum-functionalized reduced graphene (PtrGO) is formed due to the localized temperature enhancement that favors the reduction in both graphene oxide and platinum salt. In this respect, PtrGO catalysts are synthetized with improved chemical stability in acidic and alkaline environments and have a high specific area and increased electrochemical stability for the oxygen reduction reaction [28,33].

This paper proposes the synthesis of PtrGO using the microwave-assisted method in mild reaction conditions. The novelty of this approach is that it enables one to obtain different concentrations of Pt through a chemical green method, acting on the pH of the solution (acidic, neutral or basic) so that it correlates with the platinum content. Ethylene glycol (EG) was used for the dispersion and reduction process of GO and Pt precursors. The following sections present the effect of the pH value in the solution on the Pt nanoparticle morphology of the PtrGO. With the optimized pH value condition, the PtrGO presented a uniformly dispersed morphology of the Pt nanoparticles.

## 2. Materials and Methods

### 2.1. Reagents

GO used was procured from Abalonyx AS Norway with the following main characteristics: a carbon content of about 85% atomic/80% weight, a bulk density of about 9–12 g/liter, acid product, H_2_PtCl_6_ crystalline metals with a basis of 99.9999% from Alfa Aesar, ethylene glycol, 99+% spectrophotometric grade from Sigma Aldrich and NaOH from Alfa Aesar. For all the experiments, the chemicals used did not need a previous purification. The only purification that was performed was for water using the Millipore system.

### 2.2. Synthesis

**Figure 1:**
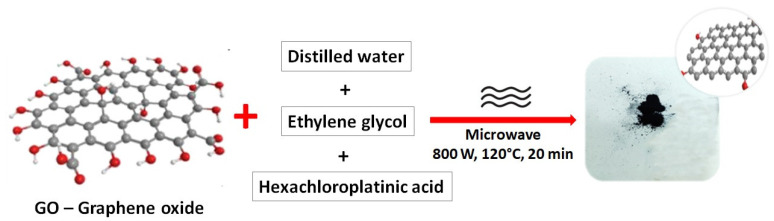
Summarized scheme of the PtrGO powder synthesis using the microwave method.

PtrGO was synthesized using the microwave-assisted method and functionalization with platinum from the H_2_PtCl_6_ precursor [30]. The prepared samples were obtained in the same conditions using the same polar solvent (ethylene glycol) but with a different pH of the solution. A suspension containing 0.25 g dry GO, 100 mL distilled water and 50 mL ethylene glycol was prepared by sonication. In Figure 1, the PtrGO powder synthesis using the microwave method is summarized. In accordance with the purpose of this work, several reaction mixtures were prepared by modifying the pH of the solution as follows. The pH of the reaction mixture was measured before its introduction into the reaction cell. Three samples were prepared by modifying the pH as follows: (i) PtrGO1 with a pH of 3.3 was obtained without NaOH; (ii) the pH of PtrGO2 was adjusted to 11.7 using 1 mL of NaOH (1M); and (iii) PtrGO3 with pH of 7.2 was prepared using 0.1 mL NaOH. The hexachloroplatinic acid solution (0.02 M) was prepared in a 50 mL volumetric flask as follows: a quantity of 0.41 g of hexachloroplatinic acid was weighed on an analytical balance, inserted into a 50 mL volumetric flask and dissolved in a quantity of distilled water before topping up with distilled water to the mark on the neck of the flask. The mixtures were immediately placed in the microwave reactor (MARS 6-CEM) for 30 min (power: 800 W and temperature: 180 °C), and the temperature was increased for 20 min. The Pt on graphene dispersions was obtained after being sonicated for 30 min. The samples were then filtered and washed first with a 200 mL mixture of 1:1 ethanol and distilled water and then with 200 mL of distilled water. The last step was to dry the samples in the oven for 8 h at 80 °C. The powder was kept in the exicator.

### 2.3. Instrumentation

FE-SEM was used to investigate the microstructure of the reduced PtrGO powder. The secondary electron (SE) mode offered information about the topography, and energy dispersive spectroscopy (EDX) analysis was used to identify the chemical elements present. The powders were placed on a double-sided sticky carbon tape and then blown with compressed air. The method by which the yield of platinum recovered from the PtrGOs catalysts was increased was to subject the samples to a mineralization/extraction with a mixture of sulfuric, perchloric and hydrofluoric acid, followed by an extraction with aqua regia. The microwave digester used was a Milestone 1200 MEGA system. In this particular study, the use of the back-scattered (BS) electron mode proved to be very efficient in terms of determining the distribution of Pt throughout the sample, since the only constituents were Pt (Z = 78) and C (Z = 6), which are two elements with very distinct atomic numbers, and, therefore, they are easily observed in this mode. The contact angle was determined using a DSA 100 Drop Shape Analyzer. To measure the surface hydrophilicity and wettability, the contact angle measurements were carried out. For this, a droplet of 5 µL of demineralized water was added to the PtrGO surfaces, and the angle formed at the liquid intersection with the solid samples was calculated using the Young–Laplace integration method. Thermogravimetric analyses (TGA) were conducted using a Netzsch STA 449F Jupiter Simultaneous Thermal Analyzer. The specific surface was determined from the measured values of the adsorption/desorption isotherm of gaseous N2 at 77 Kelvin using BET analysis (Brunauer, Emmett and Teller) with the Autosorb IQ Quantachrome equipment.. Degassing of the samples was conducted under vacuum conditions at 115 °C for 4 h. The adsorption isotherms revealed the amount of gas adsorbed at a constant temperature, increasing the pressure until the pore-filling saturation was reached. The pore size distribution and microporous volume were calculated by the BJH (Barnett–Joyner–Halenda) procedure. XRD spectra were obtained using a Rigaku MiniFlex600 powder diffractometer (P-XRD) equipped with a CuKa X-ray source at the wavelength of 1.541838 Å. Scans were collected within the 10 to 90° 2θ scanning range using a 0.01° scanning step and 1.0° min^−1^ scanning speed with a monochromator for suppressing the background noise levels. Electrochemical characterization was performed using a Princeton Applied Potentiostat/Galvanostat 2273. A 0.196 cm^2^ glassy carbon (GC) rotating disk electrode (RDE) from Origalys was used as a working electrode in a standard cell using a platinum counter electrode and a 3M Ag/AgCl reference electrode from Methrom (Ag/AgCl REF). Platinum-containing solution was prepared by overnight sonication of 5 mg catalyst powder, 240 µL of isopropyl alcohol and 20 µL of Nafion^®^ 5 wt.% solution. A quantity of 7 µL aliquot was dropped onto the clean RDE disk surface. Before each catalyst solution was deposited, the GC electrode was cleaned by soft polishing with a diamond-based compound (0.05 μm) and alumina slurry (1.0 μm). After each poshing step, the electrode was washed with demineralized water (DW). To remove the abrasive paste residues, the GC was rinsed using sonication with DW for 5 min. Cycling voltammetry (CV) measurements were recorded using a 0.5 M H_2_SO_4_ electrolyte at room temperature. Prior to each measurement, the electrolyte was purged with nitrogen for 30 min. In order to determine the number of electron transfer numbers (*n*) for the oxygen reduction reaction (ORR), linear sweep voltammograms (LSV) were measured with a 5mV/s scan speed using an oxygen-saturated electrolyte. All the potentials are reported vs. Ag/AgCl (REF electrode).

## 3. Results and Discussion

**Figure 2:**
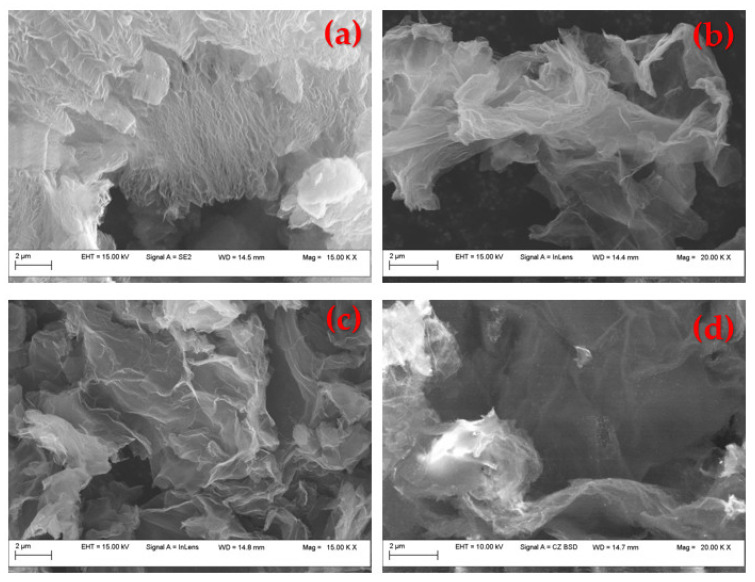
SEM micrographs of: (**a**) commercial graphene oxide; (**b**) PtrGO1; (**c**) PtrGO2; (**d**) PtrGO3.

Figure 1a–d presents the SEM images of the commercial reduced graphene oxide (rGO) in comparison with the PtrGO1, PtrGO2 and PtrGO3 samples, respectively. From Figure 1, it is observed that all the samples present a flake-like and wrinkled morphology. However, the structure of the rough reduced graphene oxide was preserved after the microwave treatment. Table 1 presents the platinum concentration calculated by atomic adsorption spectroscopy (AAS) and energy dispersive spectroscopy (EDX) [34]. For exemplification, Table 2 shows the total elemental composition of PtrGO3 determined by EDX, corresponding to Figure 2d.

The mineralization/extraction method was as follows: 2 mL H_2_SO_4_^+^, 2 mL HClO_4_^+^2 mL and HF-power program (5 min at 250 W, followed by 5 min at 400 W, 5 min at 650W and, finally, 5 min at 250 W). The vials were cooled and depressurized, and 3 mL of aqua regia was added. The determination of Pt was performed by atomic absorption spectrometry, using the flame as an atomization system. The machine used was the Varian 240FS DUO.

**Figure 3:**
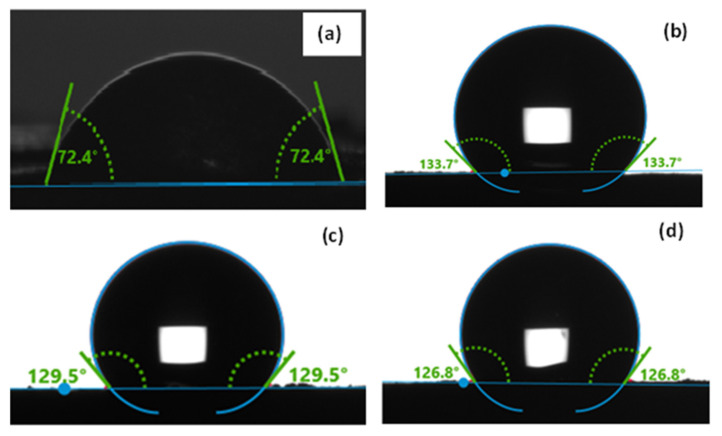
Water contact angle of: (**a**) commercial graphene oxide (GO); (**b**) PtrGO1; (**c**) PtrGO2; (**d**) PtrGO3.

Graphene and graphene oxide are hydrophilic, with a contact angle between 56° and 67° [35,36]. Moreover, reduced graphene oxide has hydrophobic characteristics as a result of a very low concentration of oxygen, in comparison with graphene oxide [37]. The calculated contact angle values for the commercial graphene oxide (GO) and PtrGO1, PtrGO2 and PtrGO3 samples are presented in Figure 3. It is observed that graphene oxide has a contact angle of 72.4° that demonstrates the hydrophilic feature of GO. Moreover, the contact angle > 90^o^ of the platinum-functionalized samples corresponds to a low level of surface wettability and high hydrophobicity, which is an important property for the further use of these materials as fuel cell catalysts. The calculated contact angle values of PtrGO1, PtrGO2 and PtrGO3 are 133.7°, 129.5° and 126.8°, respectively. These results prove that all the samples are hydrophobic and do not influence the wettability of the rGO matrix, because the contact angle is higher than 90°.

**Figure 4:**
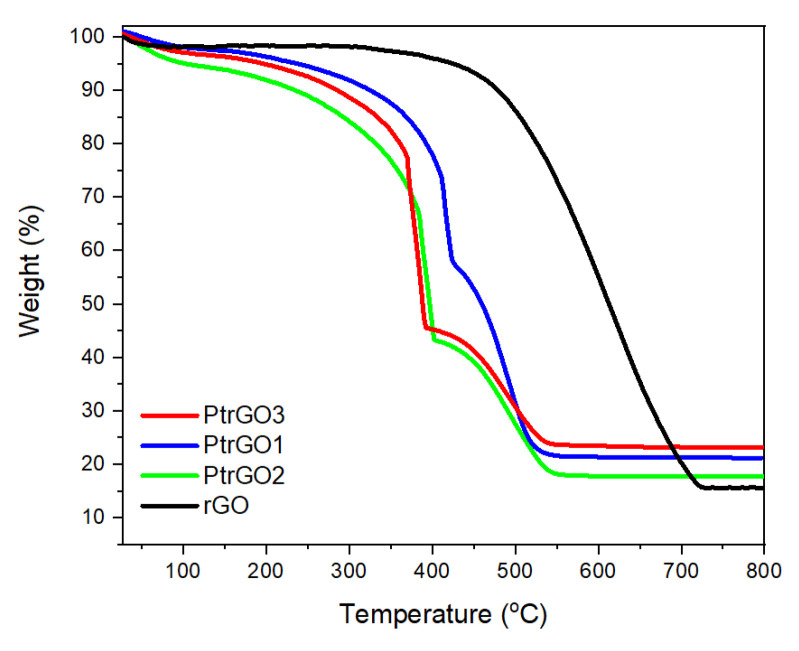
Thermogravimetric analysis of the samples of the rGO-supporting matrix with respect to the PtrGO1, PtrGO2 and PtrGO3 samples.

The thermogravimetric analysis of the commercial graphene oxide (GO) and PtrGO1, PtrGO2 and PtrGO3 samples is presented in Figure 4.

Figure 4 presents the thermogravimetric stability (TGA) analysis of the PtrGO samples against the rough reduced graphene oxide and commercial graphene oxide (GO). It is observed that GO presents three steps of decay. The first weight loss between 27 °C and 135 °C is due to the release of the trapped water molecules between the layers of GO and may be due to the moisture present in the sample. The decay of the curves between 135 and 225 °C is attributed to the loss of epoxy and hydroxyl functional groups and remaining water molecules. Between 225 and 800 °C, the samples undergo a slow and smooth weight loss, which is accredited to the thermal decomposition of stable oxygen functionalities. As carbon material cannot be oxidized or burned under inert conditions, the residual weight percentages are not stabilized. The thermogram of rGO shows almost no mass loss up to 400 °C. Above this temperature, the sample’s weight is drastically reduced to about 15 %, which represents the rGO residual mass. TGA analysis of all the PtrGO samples exhibited a mass loss in three stages. The first decay is due to the release of water at about 110 °C and the second is between 300 and 400 °C and is attributed to the losses of the epoxy and hydroxyl functional groups and remaining water molecules [29]. A significant mass loss was observed in the temperature range between 400 °C and 550 °C, representing the degradation of the reduced graphene oxide. The starting temperature for abrupt weight loss decreased with the increase in the platinum concentration, indicating a correlation between Pt and reduced graphene oxide temperature decomposition [38]. The residual platinum could not be decomposed in this range of temperatures. The difference between the rGO and the PtrGO samples’ weight loss was calculated the Pt catalyst on rGO matrix. These values are 7.59%, 5.64% and 2.18% for PtrGO3, PtrGO1 and PtrGO2, respectively, and they agree with the atomic adsorption spectroscopy (AAS) and energy dispersive spectroscopy EDX calculations.

**Figure 5:**
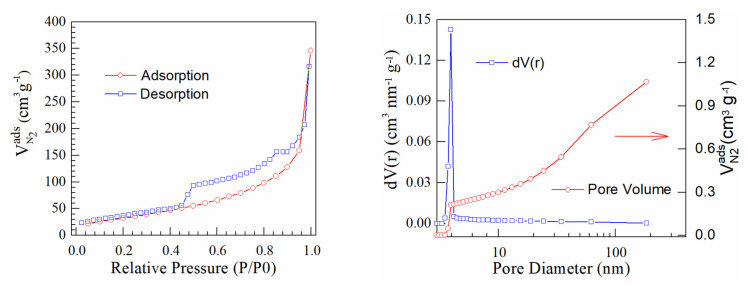
Sample PtrGO1.

**Figure 6:**
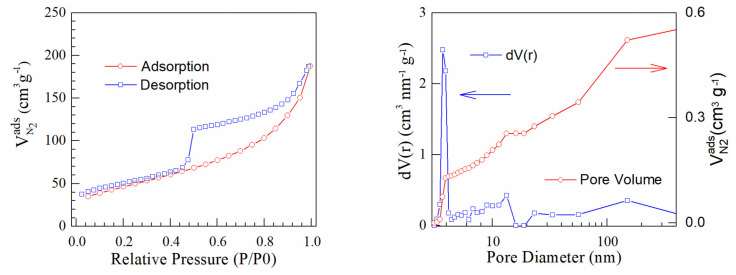
Sample PtrGO2.

**Figure 7:**
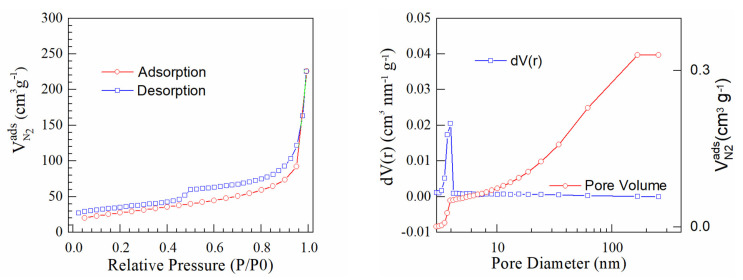
Sample PtrGO3.

Figure 5, Figure 6 and Figure 7 present BET isotherms and BJH pore size distribution curves for PtrGO1, PtrGO2 and PtrGO3, respectively. An essential feature of rGO that determines the interaction with the surface is the specific surface area [31]. rGO has an initial specific surface area of 397 m^2^/g. The specific surface areas of PtrGO are 128 m^2^/g per sample in PtrGO1, 166 m^2^/g per sample in PtrGO2 and 98 m^2^/g per sample in PtrGO3. It is observed that due to the graphene oxide platinum functionalization, all samples analyzed in this study showed a decrease in the specific surface area with increasing pH when compared to the surface area of the initial graphene oxide. Moreover, the specific surface area (m^2^/g) values presented in Table 3 agree with the ones for the platinum concentration (Table 1). It is observed that all samples analyzed in this study showed a decrease in specific surface area when compared to the surface area of the initial reduced graphene oxide. The values of the specific surface areas are very different, which might be an indication of pH influence.

The graphene sheets start to agglomerate, and the electro-crystallized form of platinum is not full. The values of the specific surface area are very different, which is an indication of pH influence.

**Figure 8:**
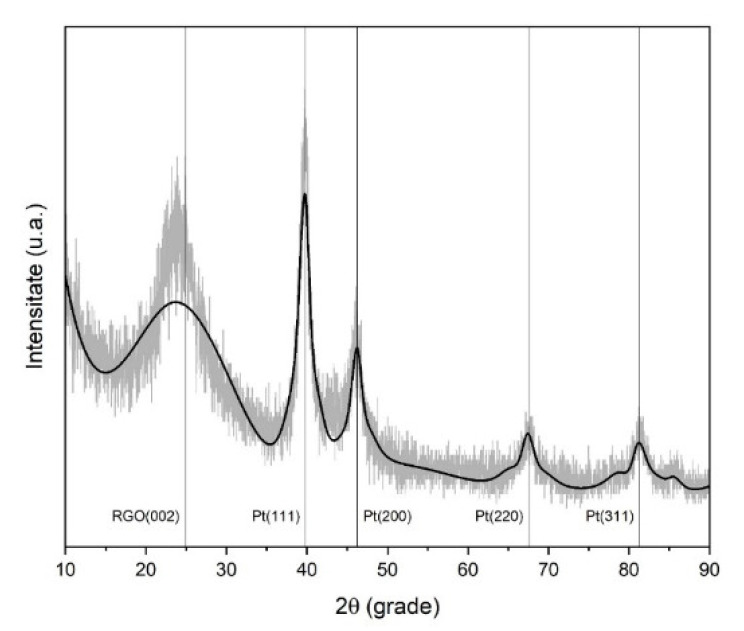
XRD spectra for Pt-decorated rGO [34].

The baseline and deconvoluted signals (Figure 8) correspond with the typical signatures for rGO (broad peak at 24.90°) and for crystalline platinum. The crystalline Pt peaks were verified using the Crystallographic Open Database (COD card number 1011113 [39]), corresponding to the cubic 225:Fm-3m space group, with a=b=c=3.92023 Å and α=β=γ=90°, for a lattice unit volume of 60.247 Å. The average crystallite size was determined at 1257 Å. Miller indices with corresponding peak parameters are given in Table 4 [17]. The SEM analysis corresponds to the microwave synthesis of platinum-decorated rGO and not a fully deposited catalytic layer. Therefore, the identification of any textural elements is not realistic, and the platinum crystallite size can be identified from the XRD data.

**Figure 9:**
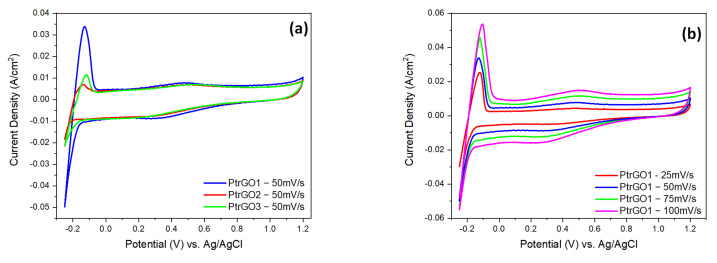

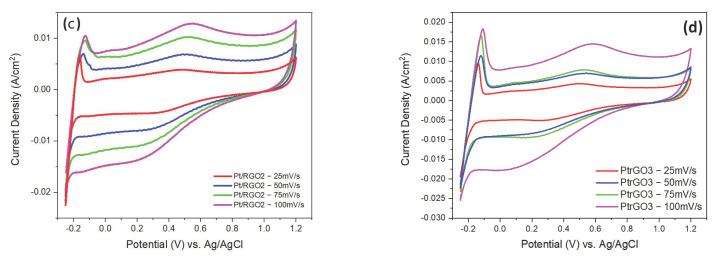
(**a**) The overlayed CVs of PtrGO1, PtrGO2 and PtrGO3 recorded with 50 mV/s in domain of − 0.25 to 1.2 V vs. Ag/AgCl; (**b**–**d**) CV of PtrGO1, PtrGO2, PtrGO3 recorded at 25 mV/s, 50 mV/s, 75mV/s and 100 mV/s scan rates.

The oxygen reduction reaction (ORR) electrocatalytic activity of PtrGO1, PtrGO2 and PtrGO3 catalyst samples was investigated by measuring the cycling voltammetry (CV) at different scan rates (25 mV/s, 50 mV/s, 75mV/s and 100 mV/s) using nitrogen-saturated electrolytes. Linear sweep voltammetry (LSV) at different RDE rotation speeds per minute (rpm) (250, 500, 750, 1000 and 1500) was performed in oxygen-saturated solution. As presented in Figure 8, the overlayed CVs of PtrGO1, PtrGO2 and PtrGO3 scanned with 50 mV/s between 0.25 and 1.2 V vs. Ag/AgCl show peaks associated with the electrochemical reactions that take place at the interface between the catalyst surface and the electrolyte. All the samples display similar behavior, and the current density value depends on the electrocatalytic activity. The peaks between −0.2 V and −0.5 V in the anodic and cathodic direction scan (Figure 9) are associated with the hydrogen adsorption/desorption on the platinum crystal. At higher potentials, in the scan in the anodic direction (0.05 V to 0.3 V), the reduced graphene oxide is slightly oxidized, and double-layer oxygenated groups cover its surface [38,39]. The peak at about 0.45 V represents platinum oxidation that continues up to 1.2 V, with platinum oxide (Pt–O) formation on the metal surface. In the reverse scan, the peak at 0.3 V represents the oxygen reduction reaction (ORR) of Pt–O on the cathode surface in a proton exchange fuel cell (PEMFC). It is known from the literature [40,41,42,43,44] that although the MW process has been used to reduce GO, the conditions used (temperature and reduction time) cannot be scaled for functionalization with Pt. The advantage of the method used in this study is the simultaneous reduction in GO with the functionalization of GO with Pt, with both processes being in situ, in a reduced time (20 min). Comparing the corresponding values of the current density, PtrGO1 has the highest catalytic activity in hydrogen adsorption/desorption, and PtrGO2 has the lowest activity in these reactions. The samples’ structure and ORR current density are influenced by the sweeping rate, as is shown in Figure 9 b–d. 

**Figure 10:**
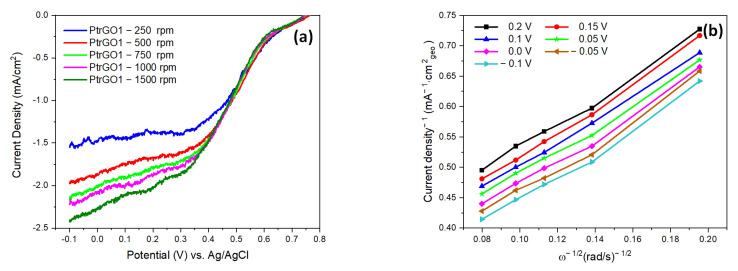

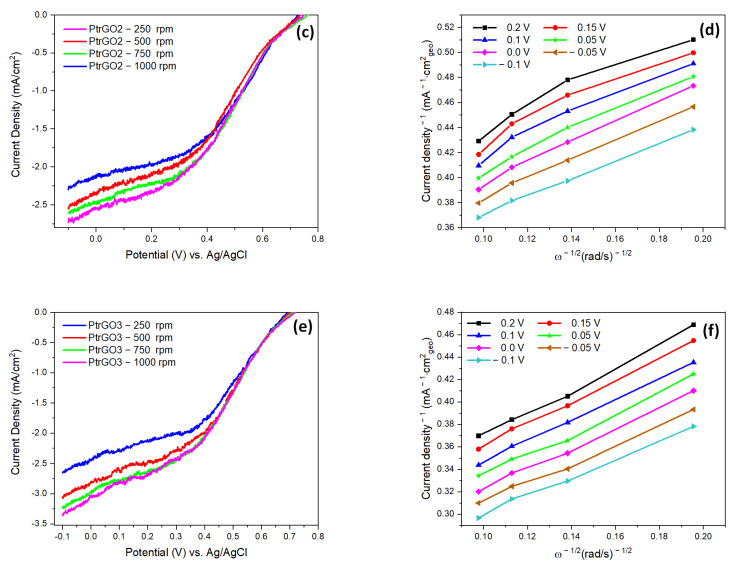
LSV plots for PtrGO1, PtrGO2 and PtrGO3 recorded at 5mV/s (**a**,**c**,**e**) and the calculated Koutecky–Levich plots (**b**,**d**,**f**).

Figure 10a,c,e presents the linear sweep voltammogram plots for PtrGO1, PtrGO2 and PtrGO3 recorded at 5mV/s between 0.8 V and −0.1 V at different RDE rotation speeds (250 to 1500 rpm). LSV measurements were used to calculate the Koutecky–Levich (K-L) plots (current density (mA^−1^ cm^2^_geo_) vs. ω^−1/2^ ((rad/s)^−1/2^)) at different potentials, between 0.2 V and −0.1 V. Figure 10b,d,f presents the current density plotted vs. ω^−1/2^ ((rad/s)^−1/2^. These graphs show a good linear relationship between the current density and RDE rotation speeds, which confirms that ORR, for all the catalysts, can be regarded as a first-order reaction [43,44]. The calculated electron transfer numbers (*n*) determined from Koutecky–Levich plots, with the linear fit in the inset, are presented in Figure 10b,d,f. They are between 3.1 and 3.8 in the potential domain of 0.2 V to −0.1 V, and these values increase with the platinum concentration determined by the pH solution containing rGO and the H_2_PtCl_6_ precursor. These values correspond to four-electron transfer oxygen reduction reactions and direct H_2_O formation during ORR [45,46] and agree with the results published in [47], in which the results obtained for Pt supported on graphene oxide (GO) and reduced graphene oxide (rGO) prepared by sonication are presented.

## 4. Conclusions

Microwave synthesis is a fast and energy-efficient approach that can be used to prepare various catalysts. Moreover, it was demonstrated that the pH of PtrGO-based dispersions influences the structural, morphological and electrochemical properties of the catalysts. Using EDX analysis, it was shown that the highest calculated the platinum concentration of 6.5 weight% corresponds to the neutral pH (7.2). SEM analysis provided an indication of the flake-like and wrinkled morphology of Pt-decorated rGO. However, the structure of the rough reduced graphene oxide was preserved after the microwave treatment. Thermogravimetric characterization proved that all the samples, including rGO, were incompletely decomposed. From the difference between the residual mass of rGO and PtrGOs, it was possible to calculate Pt loading values that agree with the AAS and EDX measurements. PrGO2 had the largest surface area (166 m^2^/g); thus, it is clear that the functionalization degree of rGO is small. Therefore, the specific area indicates that the microwave reaction did not obtain a high Pt concentration on rGO. The XRD spectrum presents a wide peak at about 2θ of 24.90°, corresponding to graphene oxide, as well as the specific peaks of 39.79°, 46.25°, 67.62° and 81.28° assigned to Pt (111), Pt (200), Pt (220) and Pt (311), respectively, which clearly indicate the presence of platinum anchored to the reduced graphene oxide lattice. Although PtGO3 had the highest concentration of Pt, we consider that the platinum particles were conglomerated and not very well-dispersed, which explains its lower electrochemical ORR performance compared to PrGO1. Moreover, in PrGO1 synthetized in an acidic environment, with 4.32 Pt (weight%) determined by EDX, the platinum was much more dispersed, which explains its better electrochemical behavior. These results confirm the influence of the pH of the solution used for synthesis in the microwave method of different platinum concentrations for reduced graphene oxide.

## Figures and Tables

**Table 1:** The average concentration of Pt by atomic adsorption and EDX [34].

Samples	pH	Pt (Wt.%) ^a^ by AAS	Pt (Wt.%) ^b^ by EDX
PtrGO1	3.3	4.1	4.3
PtrGO2	11.9	2.0	2.2
PtrGO3	7.2	6.5	5.7

^a^ Measured by atomic adsorption spectroscopy (AAS). ^b^ Measured by energy dispersive spectroscopy (EDX).

**Table 2:** The total elemental composition of PtrGO3 determined by EDX, corresponding to Figure 1d [34].

Element	Wt.%
C K	73.2
O K	21.1
Pt M	5.7
Totals	100.0

**Table 3:** BJH desorption summary [34].

Samples	Specific Surface (m^2^/g)	Pore Volume (cc/g)	Pore Radius (Å)
PtrGO1	128	0.568	18.543
PtrGO2	166	0.289	19.672
PtrGO3	98	0.329	19.667

**Table 4:** XRD peak parameters.

a/a	2ϑ (Deg.)	Miller (hkl)	d (Å)	FWHM (Deg.)	Size (Å)	Phase
1	24.90	002	3.573	4.49	18.9	rGO
2	39.79	111	2.263	1.38	64.0	Pt
3	46.25	200	1.962	1.69	53.0	Pt
4	67.62	220	1.384	2.26	44.0	Pt
5	81.28	311	1.182	1.74	63.0	Pt

## Data Availability

Not applicable.
